# More exposure to medical injury news, better doctor-patient communication, but less doctors' professional identity: A moderated chain mediation model

**DOI:** 10.3389/fpubh.2022.1045014

**Published:** 2022-12-22

**Authors:** Qiwei Li, Jie Zhou, Lei Chen

**Affiliations:** ^1^Key Laboratory of Behavioral Science, Institute of Psychology, Chinese Academy of Sciences, Beijing, China; ^2^Department of Psychology, University of Chinese Academy of Sciences, Beijing, China; ^3^Department of Ultrasound, Peking University First Hospital, Beijing, China

**Keywords:** news exposure, outgroup attribution, anxiety, doctor-patient communication, social support

## Abstract

**Objectives:**

In recent years, news of medical malignant injury events has become common in China. However, it is unclear how exposure to this news affects medical staff.

**Methods:**

The present study collected data from a sample of 311 medical staff in China. It explored the effect of exposure to such news on medical staff's communication and willingness to let their children be doctors, which was an attitude that reflects their professional identity well. In addition, this study also examined the mediating roles of outgroup attribution and anxiety, and the moderating role of social support.

**Results:**

The results showed that exposure to news of medical injury could positively and directly predict the quality of doctor-patient communication, but negatively and indirectly predict medical staff's willingness to let their children become doctors. These effects existed through the mediating role of anxiety and the chain mediating role of both outgroup attribution and anxiety. In addition, social support could mitigate the negative correlation between news exposure and outgroup attribution.

**Conclusions:**

These results suggest that news of medical malignant injury events may incentivize medical staff to improve the quality of communication in the short term, but it is not conducive to medical staff's long-term mental health. That is, exposure to news of medical injury is likely to lead to a negative influence on their professional identity, although social support can alleviate this negative influence.

## 1. Introduction

In the last 10 years, the dynamics of doctor-patient relationships in China have not been optimistic. According to a white paper on the Practice of Chinese Doctors published in 2018, 66% of doctors had experienced medical disputes of varying degrees (1). The very difficult relationships between patients and physicians have caused great concern in Chinese society.

The media, and especially media coverage, have a profound impact on doctor-patient relations (2). Social media portray almost one occurrence of violence against doctors every couple of days, which generally go viral instantly (3). Furthermore, in an effort to improve doctor-patient relations, recent news reports have tended to portray doctors as victims of vicious doctor-patient conflicts (4, 5). From the point of view of patients, some researches have confirmed that the news may improve doctor-patient trust by generating compassion for doctors (4, 6). However, few scholars have studied the influence of the coverage of medical malignant injury events on doctors' perceptions. How will such coverage affect the way doctors communicate with patients and their acceptance of the profession? Are the directions of these two effects consistent? To address these questions, we explore the effect of doctors' exposure to news of medical malignant injury events on both their communication quality and willingness to let children be doctors, and the mediation model of this effect through outgroup attribution and anxiety with the moderator of social support. And then the mechanism between doctors' exposure to medical injury news and their behavioral and psychological outcomes can be deeply analyzed so as to provide some suggestions for increasing doctors' professional health and identity and improving doctor-patient relations.

### 1.1. Superficial behavioral and deep psychological outcomes of exposure to violent coverage

We hoped to explore the influence of exposure to news of medical malignant injury events from two perspectives: superficial behavior and deep psychology. We speculated that it would be possible to obtain two opposite results.

From a behavioral perspective, we examined the effect of exposure to violent media coverage on doctor-patient communication behavior, which plays an important role in a good doctor-patient relationship. Chen et al. (7) found that doctor-patient communication was one of the top three factors that affect the doctor-patient relationships. Roter (8) employed a widely used system for coding both doctor and patient communication, which is called the Roter Interaction Analysis System (RIAS). It includes many mutually exclusive and exhaustive categories, such as biomedical and psychosocial/lifestyle questions asking about positive talk, social chit-chat, and so on. A positive correlation has been found between strong communication and patient satisfaction (9).

However, the influence of exposure to news of medical malignant injury events on doctor-patient communication is not clear at all. According to the protection motivation theory (PMT), individuals can perceive and respond to threats in their environment (10). There is no doubt that the more people are exposed to such news, the greater they perceive patients as a threat. One possibility is that this perceived threat makes it harder for medical staff to communicate in a friendly manner with patients. Another possibility is that to protect themselves from this perceived threat, medical staff will have more procedural communication with patients (such as asking about the patient's health condition and avoiding aggravating the patient), which may help to improve the quality of communication. The PMT proposes that if an individual believes the behavior can mitigate or avoid the threat and that he or she has the ability to do so, the individual will engage in a coping behavior that protects against the identified threat (10). Salmeen et al's study (11) has also empirically shown that exposure to negative media coverage of minorities will motivate minorities to engage in collective action efforts aimed at improving their situation. And many surveys found that doctors had made some defensive practices of positive communication, such as more detailed patient explanations and note-taking, in order to avoid the possibility of a patient complaining or attacking (12, 13), because the miscommunications between medical staff and patients were major inducers for violent attacks (14). Therefore, we made a hypothesis supporting the latter possibility. That is:

**Hypothesis 1**. *Medical staff who are exposed to more news of medical malignant injury events have better doctor-patient communication*.

From a psychological perspective, how exposure to violent coverage influences a doctor's professional identity should be taken into consideration, since it is worth exploring whether doctors can remain in the occupation for the long term. Professional identity is a form of social identity, including deep insight into professional performance, and the establishment of professional values and goals that are widely accepted by staff (15). Previous studies have shown that professional identity improves work enthusiasm and the quality of care, reduces turnover intention, and optimizes hospital human resource management (16, 17). According to white papers, for many years, the Chinese Medical Doctor Association has been concerned about whether doctors are willing to let their children be doctors (1). This index is a great indicator of doctors' professional identity, because it can reflect doctors' overall appreciation of their compensation, practice safety, and social reputation.

The influence of exposure to news of medical malignant injury events on medical staff's willingness to let their children be doctors may be different from its influence on their superficial communication behavior. Since the wish to guard against dangers to life for oneself and one's children is basic to human nature, the perceived threat of exposure stemming from violent coverage may make medical staff prevent their children from being doctors. According to a survey related to medical malignant injury events in 2012, 78.6% of doctors were doubtful about their choices at the time, and some of them reported that they would not allow their children to engage in clinical work (18). Exposure to news of medical malignant injury events may increase medical staff's perception of workplace violence. And the perception of workplace violence has then been found to be negatively related to medical staff's professional identity (19) and be positively correlated with their professional burnout (20). Moreover, an investigation has found that violence against doctors leads to a 0.6% decrease in the number of students enrolled in medicine-related majors, and the violence-related news reduces the quality of medical students too (21). Thus, we proposed:

**Hypothesis 2**. *Medical staff who are exposed to more news of medical malignant injury events are less willing to let their children be doctors*.

### 1.2. The mediators: Outgroup attribution and anxiety

According to attribution theory (22), human beings have a natural need to understand the causes of events, especially when the outcomes are unexpected. This is likely to be the case with medical injury events, because they are regarded as a violation of interpersonal harmony norms. Furthermore, the theory argues that groups tend to attribute negative acts committed by an outgroup member to his/her internal, dispositional factors (23). They are more likely to attribute the injuries that other groups have committed against them to others' internal characteristics (24), especially when such injuries occur frequently. Previous studies about interpersonal violence have also found that an escalation in the severity and frequency of violence tends to make victims more likely to attribute the violence to external factors such as the attackers (25). Walter et al. (26) confirmed that exposure to the information of collective victimization would heighten the perceived responsibility of the outgroup for the injuries. In addition, He (27) suggested that doctors who had recently experienced conflict with patients were more likely to view the patient as the source of the conflict. And Enosh et al. (28) found that community-based family physicians who were exposed to patients' aggression attributed to the attacker's internal locus. Therefore, we speculated that exposure to news of medical malignant injury events might make doctors more likely to attribute to the outgroup (i.e., patients).

**Hypothesis 3**. *Medical staff who are exposed to more news of medical malignant injury events are more likely to attribute these events to the outgroup*.

It has also become clear that anything that implies important, harmful consequences for the individual can generate an emotional reaction (29). And when a person experiences an intense concern that something negative will befall her/himself in the future, a feeling of anxiety will emerge (30). Exposure to medical malignant injury events increases the perceived threat posed by patients and thus naturally leads to an anxiety response in doctors (31). Previous researches regarding the outcomes of violence initiated by patients have indeed shown that it causes severe anxiety (32, 33). Therefore, we proposed:

**Hypothesis 4**. *Medical staff who are exposed to more news of medical malignant injury events are more anxious*.

Outgroup attribution means that the occurrences of these medical events are due to the patients and out of the doctors' control, which result in perceptions of low control and high risk on the doctors' part. When doctors perceive high risks with low control, they take defensive medical behaviors to avoid conflicts between themselves and their patients (27), which may influence their communication style (34). And the findings are inconsistent. Some studies have suggested that doctors may not communicate frankly with the patient regarded as a potential threat, or even take threatening patients off their lists (27, 34). But these behaviors happen more often to the doctors with high control. For the doctors perceiving high risks with low control (e.g., the medical injuries are completely attributed to patients), some other studies have supported the theory that doctors may communicate with their patients and families more effectively and compassionately at every encounter, helping them understand clinical recommendations and so on (35). Thus, we hypothesized that exposure to medical injury events might lead to outgroup attribution by doctors, making them defensive with low control and improving their communication quality. After all, the best care is the best defense (36). Therefore, we proposed:

**Hypothesis 5**. *Outgroup attribution mediates the positive relationship between medical staff* '*s exposure to news of medical malignant injury events and their quality of communication*.

Similar to the role of outgroup attribution, anxiety is also a sign of high-risk perceptions and a low sense of control (37). When people perceive a threat, they become anxious and attempt to restore control, which may cause them to be hypervigilant and take some defensive behaviors (38). Wohl and Branscombe (39) have found that reminders of historical victimization initiate anxiety and then result in ingroup defensive responses. Therefore, we also hypothesized that the anxiety caused by exposure to medical injury events might improve doctors' communication quality, and proposed:

**Hypothesis 6**. *Anxiety mediates the positive relationship between medical staff's exposure to news of medical malignant injury events and their quality of communication*.

On the contrary, although the greater injury risk and lower sense of control caused by outgroup attribution may create incentives for doctors to communicate better, it will also no doubt increase their psychological burden. Therefore, outgroup attribution can affect individual career decision-making self-efficacy (40), career certainty, and professional development (41). For example, Liu et al. (42) found that lack of control negatively affected the professional identity of nurses. Previous studies have also confirmed that workplace violence reduces medical staff's sense of control over their work (43), and finally leads to turnover intentions (44). Therefore, we proposed:

**Hypothesis 7**. *Outgroup attribution mediates the negative relationship between medical staff's exposure to news of medical malignant injury events and their willingness to let their children be doctors*.

In addition, many studies have found that anxiety is negatively correlated with professional identity (45, 46). Violence increases medical staff's anxiety (47), and thus increases their turnover intentions (48). Therefore, we proposed:

**Hypothesis 8**. *Anxiety mediates the negative relationship between medical staff's exposure to news of medical malignant injury events and their willingness to let their children be doctors*.

Moreover, it can be argued that affective reactions depend on the interpretation and labeling of cognitive structures, and they are inseparable (49). Some theorists have suggested that blaming others means admitting weak personal control in the attribution of causality, and these processes serve the function of reducing an individual's sense of control over their environment and perceived avoidability (50), which may cause inevitable anxiety (37, 51). Furthermore, empirical research also indicates that anxiety is significantly greater in “blame-others” victims than in “self-blame” victims (52, 53). Therefore, we proposed:

**Hypothesis 9**. *Outgroup attribution and anxiety have a chain mediating effect on the relationship between medical staff's exposure to news of medical malignant injury events and their quality of communication*.

**Hypothesis 10**. *Outgroup attribution and anxiety have a chain mediating effect on the relationship between medical staff's exposure to news of medical malignant injury events and their willingness to let their children be doctors*.

### 1.3. The moderator: Social support

Typically, people want to talk with others about stressful life events due to their basic needs for belonging and love (54). An individual sharing stress-related thoughts and feelings with members of their social network reflects their attained social support (55). Social support may help to buffer people from the negative effects of violence exposure through a variety of mechanisms, such as reducing the perceived threat and enhancing their sense of control (56, 57). Thus, social support is a process used to gain security. Past researchers have theorized that peers of accident perpetrators tend to externalize accident responsibility, lest they be blamed for similar accidents (58). Additionally, the security gained from social support may help people to interpret and explain medical injury events more objectively, combining internal and external attributions (59) rather than only focusing on outgroup attributions. This suggests that social support may moderate the relationship between exposure and outgroup attribution.

Furthermore, social support is considered as a social resource. Individuals who possess social support view themselves as valued and worthy of love and appreciation, and they have been found to demonstrate better resilience and mental health in the face of stressful situations (60). Previous studies have proven that social support can buffer the association between various acts of violence and anxiety (61, 62). Bourne et al. (63) also found that the more often doctors spoke to their colleagues, the less likely they were to suffer from anxiety. Therefore, we proposed hypotheses 11 and 12:

**Hypothesis 11**. *Social support moderates the relationship between medical staff's exposure to news of medical malignant injury events and their outgroup attribution. Specifically, the association between exposure and outgroup attribution is weakened for doctors with high social support*.

**Hypothesis 12**. *Social support moderates the effect of medical staff's exposure to news of medical malignant injury events on their anxiety. Specifically, the association between exposure and anxiety is weakened for doctors with high social support*.

**Figure 1:**
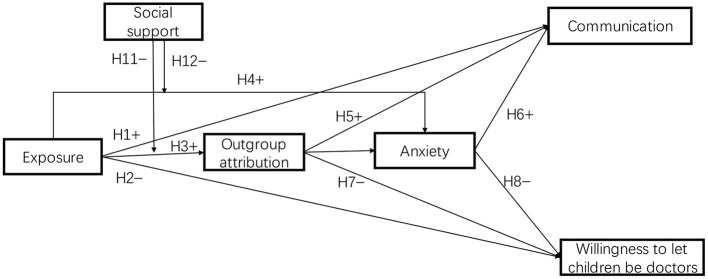
Conceptual model.

In summary, the theoretical model of this paper is shown in Figure 1.

## 2. Methods

### 2.1. Participants and procedures

The study adopted convenient sampling method and online questionnaire to investigate medical staff working in Chinese hospitals in August 2021. After reading the informed consent, participants completed the questionnaire anonymously. Assuming a moderate size of correlations (*r* = 0.3) among variables, a priori power analysis *via* the Monte Carlo simulation method was conducted to estimate the required sample size in testing a serial mediator model. It was estimated that a sample of 300 participants would provide 88% of statistical power (64). A total of 311 medical staff (see Table 1) including 102 males were finally recruited by an online questionnaire.

**Table 1:** Demographic information.

		**n (%)**
Gender	Male	102 (32.8%)
	Female	209 (67.2%)
Age	<30	57 (18.3%)
	31–40	112 (36.0%)
	41–50	42 (13.5%)
	51–60	43 (13.8%)
	>60	57 (18.3%)
Professional title	Chief/senior	46 (14.8%)
	Associate chief/senior	50 (16.1%)
	Intermediate/in charge	135 (43.4%)
	Junior	80 (25.7%)
Hospital grade	General hospital	245 (78.8%)
	Regional hospital	52 (16.7%)
	Community hospital	14 (4.5%)
Department	Clinic	212 (68.2%)
	Medical detection	70 (22.5%)
	Management	29 (9.3%)

### 2.2. Measures

#### 2.2.1. Exposure to news of medical malignant injury events

The frequency of browsing news of violent events against medical staff was measured by the question “How often did you read news of malignant medical injuries in the past 2 years?” Participants responded on a five-point scale ranging from 1 (not at all) to 5 (very often).

#### 2.2.2. Outgroup attribution

The attributional style was measured by the question “In general, to what extent are the patients primarily responsible for injuries to doctors?” Participants responded on a five-point scale ranging from 1 (not at all) to 5 (very much).

#### 2.2.3. Anxiety

Nine items adapted from Li and Li (65) assessed the anxiety felt when participants thought of violent events against medical staff (e.g., “I feel nervous and anxious”). Participants responded on a five-point scale ranging from 1 (not at all) to 5 (very much), with a higher average score indicating more anxiety (α = 0.877).

#### 2.2.4. Social support

Seven items adapted from the Social Support Revalued Scale (66) assessed the extent of social support, including 5 single-choice questions (e.g., “How much support and care have you received from family members?”), and 2 multiple-choice questions (e.g., “In the past, what sources of comfort and care did you get when you were in urgent situations?”). The single-choice questions were responded on a five-point scale ranging from 1 (not at all) to 5 (very much), and the multiple-choice questions are scored according to the number of options chosen. The scores for all questions were summed and ranged from 5 to 45, with a higher score indicating greater social support (α of the total scale was 0.704).

#### 2.2.5. Doctor-patient communication

According to the Roter interaction analysis system (8), we developed a four-item scale for doctor-patient communication quality assessment (e.g., “When you communicate with patients, will you express consent, listening, affirmation, praise, etc., such as ‘you did the right thing' and ‘well, yes, I know'?”). Participants responded on a five-point scale ranging from 1 (not at all) to 5 (very often), with a higher average score indicating higher communication quality (α = 0.810).

#### 2.2.6. Willingness to let children be doctors (index of professional identity)

This was measured by the question “Would you like your child to study medicine in the future?” Participants responded on a five-point scale ranging from 1 (not at all) to 5 (very much).

#### 2.2.7. Control variables

The control variables included gender, age, professional title, departments, and the grade of their hospitals.

### 2.3. Statistical analyses

The SPSS software package was used to organize and clean the dataset, as well as to generate the descriptive statistical analysis and correlations.

In order to test our hypothesized model (see Figure 1), data were analyzed *via* path analysis models in the Lavaan package (67); (version 0.6–9) in R (version 3.3.0). Path analysis modeling was performed using Maximum Likelihood (ML) as the estimator. Model fit was accessed by multiple fit indices, including: ratio of the chi-square to its degree of freedom, comparative fit index (CFI), goodness-of-fit index (GFI), root-mean-square error approximation (RMSEA) and standardized root mean square residual (SRMR) (68, 69). And a bootstrapping method with 5,000 resamples was used to test the indirect and direct effects in the path analysis models.

After testing the hypothesized model, adjustments were made to improve fit by eliminating paths that were not statistically significant so that the most parsimonious model that was empirically and theoretically justified was found finally. The readjusted models were then retested by using the same procedures of above path analysis modeling.

## 3. Results

### 3.1. Preliminary analyses

The means, standard deviations, and bivariate correlations of all study variables are shown in Table 2. Gender, age, professional title, departments and the grade of hospital were included as covariates in subsequent analyses.

**Table 2:** Means, standard deviations, and bivariate correlations for all study variables.

	***M***	**SD**	**1**	**2**	**3**	**4**	**5**	**6**	**7**	**8**	**9**	**10**
1. Gender	-	-										
2. Age	2.78	1.39	0.04									
3. Professional title	2.80	0.99	0.08	−0.67^***^								
4. Departments	1.26	0.53	−0.06	0.34^***^	0.05							
5. The grade of hospital	1.41	0.66	0.13^*^	0.12^*^	0.01	0.08						
6. Exposure	3.65	0.77	−0.05	0.04	−0.02	−0.09	−0.06					
7. Social support	25.88	5.34	0.11^†^	0.21^***^	−0.25^***^	0.03	0.05	0.11^†^				
8. Outgroup attribution	4.16	0.87	0.04	−0.16^**^	−0.03	−0.26^***^	−0.06	0.19^**^	0.08			
9. Anxiety	4.14	0.65	0.05	0.06	−0.17^**^	−0.21^***^	−0.07	0.36^***^	0.12^*^	0.43^***^		
10. Communication	3.86	0.75	0.04	0.25^***^	−0.19^**^	0.05	−0.14^*^	0.28^***^	0.21^***^	0.07	0.30^***^	
11. Willingness of children to be physicians	2.68	1.16	−0.03	0.23^***^	−0.14^*^	0.14^*^	0.06	0.01	0.21^***^	−0.11	−0.17^**^	0.04

† *p* < 0.1.

* *p* < 0.05.

** *p* < 0.01.

*** *p* < 0.001.

Notably, exposure to news of medical malignant injury events was positively correlated with outgroup attribution (*r* = 0.19; *p* = 0.001) and anxiety (*r* = 0.36; *p* < 0.001), supporting Hypotheses 3 and 4. Furthermore, it was positively correlated with the quality of communication (*r* = 0.28; *p* < 0.001), consistent with Hypothesis 1. However, it was not significantly related to medical staff's willingness (*r* = 0.01; *p* = 0.89) to let their children be doctors; therefore, Hypothesis 2 was not supported.

Additionally, outgroup attribution was significantly correlated with anxiety (*r* = 0.43; *p* < 0.001). And anxiety was positively correlated with communication quality (*r* = 0.30; *p* < 0.001) and negatively correlated with willingness (*r* = −0.17; *p* = 0.003).

### 3.2. Structural model testing

Controlling the covariates, we used the Lavaan package (version 0.6–9) in R (version 3.3.0) to test the model with the Bootstrap method. The initial test of the hypothesized model (see Figure 1) yielded a poor fit (χ2(11) = 50.028, CFI = 0.851, GFI = 0.998, RMSEA = 0.107, SRMR = 0.050). Outgroup attribution did not predict the quality of communication (β = −0.03, se = 0.05, p = 0.69) and doctors' willingness to let their children be doctors (β = 0.01, se = 0.08, p = 0.88), hypothesis 5 and 7 were thus not supported. Meanwhile, the interaction variable of exposure × social support was not a significant predictor of anxiety (β = 0.04, se = 0.01, p = 0.46). Hypothesis 12 was not supported either.

**Figure 2:**
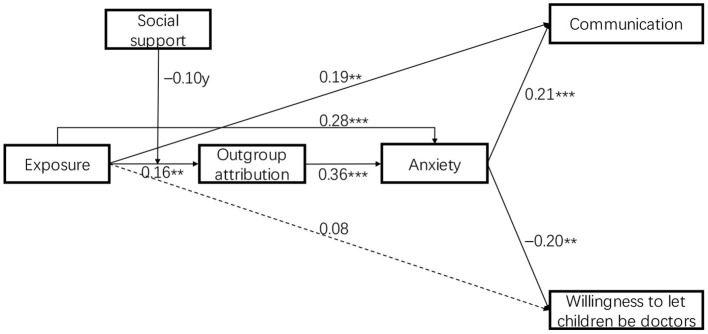
Estimation of final moderated mediation model.^†^*p* < 0.1, **p < 0.01, and ***p < 0.001.

Therefore, to adjust the model for improving the fit, above three paths that were not statistically significant were eliminated in the SEM analysis. Consequently, the model fit indices were improved greatly (χ2(8) = 19.436, CFI = 0.952, GFI = 0.971, RMSEA = 0.068, SRMR = 0.024). This final model with standardized estimates of path coefficients is presented in Figure 2.

The final SEM model suggested that exposure to news of medical malignant injury events positively predicted the quality of communication (β = 0.19, *se* = 0.06, *p* = 0.001), supporting Hypothesis 1. But the path coefficient between exposure to medical injury news and willingness to let children be doctors was not significant (β = 0.08, *se* = 0.10, *p* = 0.23). Hypothesis 2 was not supported. In addition, exposure to such news could affect these two outcome variables through the mediating role of anxiety and the chain mediating role of both outgroup attribution and anxiety. Specifically, exposure to medical injury news positively predicted outgroup attribution (β = 0.16, *se* = 0.06, *p* = 0.002), supporting Hypothesis 3. Exposure also positively predicted anxiety (β = 0.28, *se* = 0.04, *p* < 0.001), supporting Hypothesis *4*. At the same time, the path coefficient from outgroup attribution to anxiety was 0.36 (*se* = 0.04, *p* < 0.001), indicating that outgroup attribution had a significant positive impact on anxiety. Furthermore, anxiety positively predicted the quality of communication (β = 0.21, *se* = 0.07, *p* < 0.001) while negatively predicted willingness to let children be doctors (β = −0.20, *se* = 0.10, *p* < 0.001), indicating that anxiety had a significant positive mediating effect between exposure to medical injury news and doctors' communication, and a significant negative mediating effect between exposure to medical injury news and willingness to let children be doctors. Hypothesis 6 and 8 were therefore verified. And outgroup attribution and anxiety had a significant chain mediating effect between exposure to medical injury news and doctors' communication and willingness to let children be doctors, supporting Hypothesis 9 and 10. Results of Bootstrap = 5,000 in Table 3 showed that these indirect effects mentioned above were all significant.

**Table 3:** Mediating effect and 95% confidence interval estimated by the Bootstrap method.

**Path**		**Indirect effect estimation**	**CI at 95% level**	
Ex → Co: Total effect		0.265	0.151	0.369
Indirect effect	Ex → An → Co	0.059	0.025	0.105
	Ex → OA → An → Co	0.012	0.004	0.030
Ex → Wi: Total effect		0.008	−0.163	0.193
Indirect effect	Ex → An → Wi	−0.056	−0.153	−0.036
	Ex → OA → An → Wi	−0.012	−0.044	−0.006

CI, confidence interval; Ex, exposure to news of medical malignant injury events; OA, outgroup attribution; An, anxiety; Co, communication; Wi, willingness to let children be doctors.

**Figure 3:**
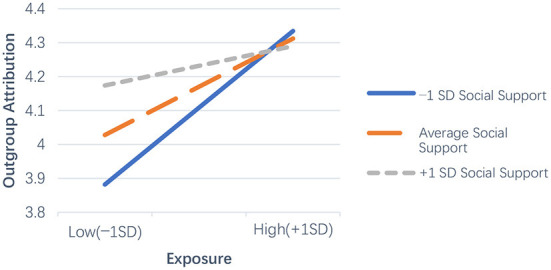
Graphical representation for moderating effect of social support on the relationship between exposure and outgroup attribution.

Besides, the analysis also showed that the interaction variable of exposure × social support was a marginally significant predictor of outgroup attribution (β = −0.10, *se* = 0.01, *p* = 0.097). To probe and visualize the interaction, we plotted the slopes of interaction at the values of social support corresponding to one standard deviation above and below the mean, while setting the covariates to their sample means. As shown in Figure 3, simple slope analyses revealed that exposure was positively related to outgroup attribution when social support was low (*simple slope* = 0.29, *se* = 0.08, *t* = 3.63, *p* < 0.001) or average (*simple slope* = 0.18, *se* = 0.06, *t* = 2.97, *p* = 0.003), but was not correlated with outgroup attribution when social support was high (*simple slope* = 0.08, *se* = 0.09, *t* = 0.83, *p* = 0.408). Hypothesis 11 was thus supported.

Then, we analyzed the moderated chain mediating effect under different conditions of social support. The bootstrapping results showed that the indirect effect of exposure on the quality of communication through outgroup attribution and anxiety was significant at low levels of social support [effect = 0.020, 95% CI = (0.006, 0.047)], but not significant in the condition of high social support [effect = 0.005, 95% CI = (−0.005, 0.023)]. Similarly, the indirect effect of exposure on willingness to let children be doctors through outgroup attribution and anxiety was also significant at low levels of social support [effect = −0.019, 95% CI = (−0.064, −0.011)], but not significant when social support was high [effect = −0.005, 95% CI = (−0.034, 0.007)]. The moderated chain mediation model in Figure 2 was verified. The results of the hypothesis testing are presented in Table 4.

**Table 4:** Results of hypothesis testing.

**Hypotheses**	**Results**
**Hypothesis 1(H1):** *Medical staff who are exposed to more news of medical malignant injury events have better doctor-patient communication*.	Supported
**Hypothesis 2(H2):** *Medical staff who are exposed to more news of medical malignant injury events are less willing to let their children be doctors*.	Not supported
**Hypothesis 3(H3):** *Medical staff who are exposed to more news of medical malignant injury events are more likely to attribute these events to the outgroup*.	Supported
**Hypothesis 4(H4):** *Medical staff who are exposed to more news of medical malignant injury events are more anxious*.	Supported
**Hypothesis 5(H5):** *Outgroup attribution mediates the positive relationship between medical staff's exposure to news of medical malignant injury events and their quality of communication*.	Not supported
**Hypothesis 6(H6):** *Anxiety mediates the positive relationship between medical staff's exposure to news of medical malignant injury events and their quality of communication*.	Supported
**Hypothesis 7(H7):** *Outgroup attribution mediates the negative relationship between medical staff's exposure to news of medical malignant injury events and their willingness to let their children be doctors*.	Not supported
**Hypothesis 8(H8):** *Anxiety mediates the negative relationship between medical staff's exposure to news of medical malignant injury events and their willingness to let their children be doctors*.	Supported
**Hypothesis 9(H9):** *Outgroup attribution and anxiety have a chain mediating effect on the relationship between medical staff's exposure to news of medical malignant injury events and their quality of communication*.	Supported
**Hypothesis 10(H10):** *Outgroup attribution and anxiety have a chain mediating effect on the relationship between medical staff's exposure to news of medical malignant injury events and their willingness to let their children be doctors*.	Supported
**Hypothesis 11(H11):** *Social support moderates the relationship between medical staff's exposure to news of medical malignant injury events and their outgroup attribution. Specifically, the association between exposure and outgroup attribution is weakened in medical staff with high social support*.	Supported
**Hypothesis 12(H12):** *Social support moderates the effect of medical staff's exposure to news of medical malignant injury events on their anxiety. Specifically, the association between exposure and anxiety is weakened in medical staff with high social support*.	Not supported

## 4. Discussion

The present research aimed to explore how exposure to news of medical malignant injury events influences doctors' superficial behavior (i.e., communication with patients) and deep psychology (i.e., professional identity) in opposite directions. Regarding the impact of exposure on doctors' communication behavior, we found that most medical staff indeed read violent news in daily life (61.7% of them chose “often” or even “very often”, *M* = 3.65), and the more often medical staff read violent news, the more positive their communication. Previous research has shown that the more doctors ask for patients' information and use more positive language, the higher the satisfaction the patient feels (9). This seems to indicate that frequent exposure to news of medical malignant injury events does not reduce the quality of doctor-patient communication, and thus may not impair patient satisfaction and doctor-patient relations either. However, we also found that the positive effect of exposure to violent news on doctor-patient communication was significantly mediated by anxiety, and serially mediated by outgroup attribution and anxiety. This means that the more medical staff are exposed to violent news, the more likely they will blame the patient and, thus, be more anxious. In addition, exposure to violent news was found to directly increase anxiety. These findings are consistent with previous reports that violence can affect the cognitions and emotions of medical staff (70), and reflect that under the perceived threat of frequent violence, medical staff have a strong sense of potential insecurity and low control. In order to protect themselves, they may hide their true emotions and adopt more procedural and normative communication, such as asking about the physical and psychological states of patients. This kind of normative communication may not show any damage to patient satisfaction and the doctor-patient relationships in the short term, but it is not a healthy doctor-patient relationship per se.

Regarding the effect of exposure to violent news on doctors' professional identity, the stress and negative emotions with long-term exposure to such news will lead to decreased job satisfaction (71), increased absenteeism, intention to quit work (72), and decreased quality of patient care (73). Our findings also provide experimental evidence demonstrating that medical staff's exposure to news of medical malignant injury events negatively predicts their willingness to let their children be doctors, which is significantly mediated by anxiety, and serially mediated by outgroup attribution and anxiety too. More importantly, exposure to news of medical injury events was found to directly affect the quality of doctors' communication, but to indirectly affect their willingness to let their children be doctors, which did not support our hypothesis 2. It may suggest that the latter involves more long-term effects. That is, exposure to violent news might have a potentially terrible influence on the professional identity of existing doctors.

Meanwhile, the current study also found that outgroup attribution did not affect doctors' communication behavior and psychological identity directly, which did not support our hypothesis 5 and 7. However, outgroup attribution could affect doctors' communications and willingness to let children be doctors through anxiety. This supports the idea that our cognitive structure can affect our emotions and, therefore, our behaviors (49). And emotion is a much closer predictor of behavior than cognition (74). Furthermore, social support had a significant moderating effect on the relationship between exposure and outgroup attribution. This suggests that adequate social support can reduce medical staff's tendency to blame medical events on patients, thereby reducing their anxiety, which contributes to long-term and friendly doctor-patient relationships. Usually, social support has been considered as one of the fundamental protective factors that buffer individuals from risk and threat. It can help to reduce medical staff's perceived stress (75) and increase their subjective happiness (76). Therefore, we should pay more attention to medical staff and offer them more social support and psychological assistance. Hospitals should establish effective communication channels with doctors, understand the difficulties they encounter in work and life, and give them timely help. In addition, hospitals can also provide psychological counseling and psychological guidance for doctors, so as to prevent or reduce doctors' anxiety and job burnout.

Moreover, we found that anxiety directly mediated the relationship between exposure to violent news and doctors' communication behavior and psychological identity. This suggests that, compared to outgroup attribution, anxiety is a more direct antecedent variable of doctors' communications and willingness to let children be doctors. However, social support did not moderate the relationship between exposure and anxiety, which did not support our hypothesis 12. This may suggest that the emotional impact of exposure to medical injury events is so large, immediate, and hard to avoid, that even social support cannot mitigate it effectively. These results imply that, besides social support from families, friends and hospitals, the government should vigorously plan and set up emergency disposal systems and modern medical systems in a legal environment, reducing medical injury events at the source. At the same time, when a medical injury event occurs, the media should report it objectively, avoiding tarnishing the images of physicians or patients blindly. On the contrary, the government and the media should increase the propagation and publicity of positive examples of good doctor-patient relationships so that doctors can feel respected and appreciated.

## 5. Limitations and future research

A pertinent limitation of this study was the cross-sectional design, which precluded inferences of the causal relationship between exposure to news of medical malignant injury events and medical staff's psychology and behaviors. Future studies can try to manipulate the medical staff's exposure by pushing relevant news regularly, increase social support they get, and intervene their outgroup attribution or anxiety to test the casual effects of our moderated chain mediation model. Besides, the self-reported measurements of all study variables may induce some bias and possibly exaggerate the medical staff's reports related to communications. Future research can use observational methods to attain the quality of doctor-patient communication or collect more objective data such as average consultation time and satisfaction rate by patients in a field study.

## 6. Conclusions

The present research provided evidence that exposure to news of medical malignant injury events can positively affect medical staff's communication in the short term, but negatively affect their willingness to let their children become doctors through the mediating roles of outgroup attribution and anxiety. In addition, social support can reduce the negative impact of exposure on outgroup attribution, which means that medical staff with a high level of social support are less likely to attribute medical malignant injury events to patients even if they have read a lot of violent news, hence leading to better and healthier doctor-patient relations.

## Data availability statement

The raw data supporting the conclusions of this article will be made available by the authors, without undue reservation.

## Ethics statement

The studies involving human participants were reviewed and approved by Ethics Subcommittee of Institute of Psychology, Chinese Academy of Sciences. Written informed consent for participation was not required for this study in accordance with the national legislation and the institutional requirements.

## Author contributions

QL and JZ conceived and designed the study and contributed to the manuscript writing and data analysis. LC conceived and designed the study and contributed to the data collection. All authors have read and agreed to the published version of the manuscript.
